# Comparative evaluation of machine learning models for predicting PD-L1 high expression in resectable NSCLC: a dual-center study integrating [^18^F]FDG PET/CT and clinicopathological features

**DOI:** 10.3389/fimmu.2026.1834643

**Published:** 2026-06-15

**Authors:** Jiong Lin, Xin Li, Jiming Tang, Haijie Xu, Xirui Lin, Chaoquan He, Peishen Li, Jiayin Wu, Weixing Huang, Hansheng Wu

**Affiliations:** 1 Department of Thoracic Surgery, The First Affiliated Hospital of Shantou University Medical College, Shantou, China; 2 Shantou University Medical College, Shantou, China; 3 Department of Gastrointestinal Surgery, The First Affiliated Hospital of Shantou University Medical College, Shantou, China; 4 Department of Thoracic Surgery, Guangdong Provincial People’s Hospital (Guangdong Academy of Medical Sciences), Southern Medical University, Guangzhou, China; 5 Joint Cardiac Surgery Center, The First Affiliated Hospital of Shantou University Medical College, Shantou, China

**Keywords:** machine learning, neoadjuvant chemoimmunotherapy, nomogram, non-small cell lung cancer, PD-L1, PET/CT

## Abstract

**Background:**

Accurate prediction of programmed death-ligand 1 (PD-L1) high expression (tumor proportion score [TPS] ≥50%) is important for identifying patients with resectable non-small cell lung cancer (NSCLC) who may benefit from neoadjuvant chemoimmunotherapy (nCIT). This study aimed to evaluate eight machine learning (ML) algorithms and develop a non-invasive, [^18^F]FDG PET/CT-based predictive model.

**Methods:**

A retrospective, dual-center cohort of 269 patients with stage IB–IIIB resectable NSCLC who underwent [^18^F]FDG PET/CT for initial staging was enrolled (training set: n=216; independent external validation set: n=53). The reference standard for PD-L1 status was immunohistochemistry (IHC) using the 22C3 assay, with TPS ≥50% defined as high expression. Baseline clinical-pathological features and the primary tumor maximum standardized uptake value (SUVmax) were extracted. Feature dimension reduction was performed using LASSO regression. Eight ML algorithms were trained and evaluated using 1000-fold bootstrap internal validation and external validation. Pairwise model comparisons of the area under the receiver operating characteristic curve (AUC) were conducted using DeLong’s test with Bonferroni correction. A two-tailed adjusted P < 0.05 was considered statistically significant. A quantitative nomogram was subsequently constructed based on the optimal parsimonious model.

**Results:**

Of the 269 enrolled patients, 79.9% were male and 32.0% were older than 65 years. LASSO regression identified five core predictors: smoking status, histological type, T stage, histological grade, and SUVmax. In the independent external validation set, Support Vector Machine (SVM) (AUC = 0.858), Random Forest (RF) (AUC = 0.849), and Logistic Regression (LR) (AUC = 0.833) demonstrated good discriminative performance. However, DeLong’s test indicated no statistically significant advantage of the complex models over the traditional LR model (all adjusted P = 1.000). Prioritizing model transparency and interpretability, an LR-based nomogram was established, which exhibited favorable calibration and provided clinical net benefit across a wide range of threshold probabilities in both cohorts.

**Conclusions:**

We developed and validated an interpretable, [^18^F]FDG PET/CT-based nomogram integrating SUVmax and clinical-pathological features to non-invasively predict PD-L1 high expression in resectable NSCLC. This study suggests that traditional LR offers comparable predictive accuracy to complex ML algorithms, while providing enhanced clinical transparency and a potential non-invasive adjunct for personalized nCIT decision-making.

## Graphical abstract

**Figure:**
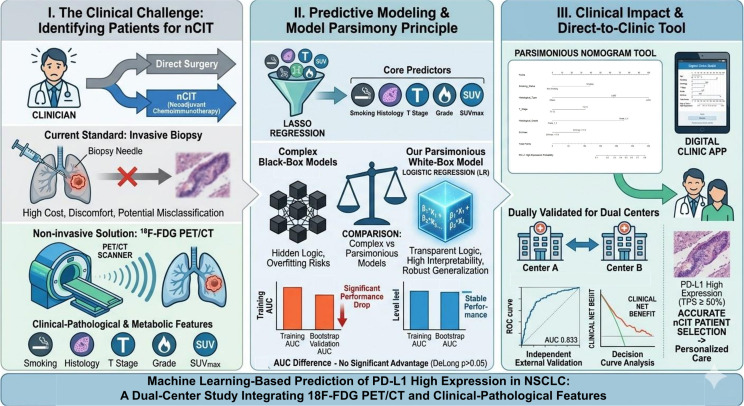

## 1 Introduction

Lung cancer remains one of the most devastating malignancies globally, with its incidence and mortality rates consistently ranking first among all cancers over the past decade (1). Non-small cell lung cancer (NSCLC) is the most prevalent histological subtype, accounting for approximately 80%–85% of all lung cancer cases (1, 2). Despite continuous advancements in therapeutic modalities, surgical resection remains the main curative treatment for resectable NSCLC (3). However, high disease heterogeneity coupled with elevated rates of local recurrence and distant metastasis result in a suboptimal 5-year overall survival (OS) rate for these patients (4). Therefore, identifying reliable biomarkers to precisely screen treatment-benefiting populations and developing personalized therapeutic regimens are important for improving the prognosis of patients with resectable NSCLC.

The advent of immunotherapy and targeted therapy over the last decade has revolutionized the lung cancer treatment landscape, significantly enhancing patient survival benefits (5, 6). Immune checkpoint inhibitors (ICIs), particularly those targeting the programmed death-1 (PD-1)/programmed death-ligand 1 (PD-L1) signaling pathway, have been widely adopted in clinical practice (7, 8). Findings from the CheckMate 816 study demonstrated that neoadjuvant chemotherapy combined with nivolumab, a PD-1 inhibitor, significantly improved clinical outcomes in patients with resectable NSCLC, with a 5-year OS rate of 65.4% (9). Numerous studies have confirmed that PD-L1 expression status is a key biomarker for predicting the efficacy of ICI therapy (10, 11). In the Phase III KEYNOTE-024 trial, treatment-naïve patients with advanced NSCLC, wild-type EGFR/ALK and a PD-L1 tumor proportion score (TPS) ≥ 50%, achieved significantly superior progression-free survival (PFS) and OS with pembrolizumab monotherapy compared to platinum-based chemotherapy (12). This predictive value has also been validated in the resectable NSCLC population: the ChiCTR-OIC-17013726 trial indicated that stromal PD-L1 expression levels were significantly correlated with pathological responses to neoadjuvant therapy (13), and the IMpower010 trial further confirmed that patients with completely resected Stage II-IIIA NSCLC and a TPS ≥ 50% derived significantly greater OS and disease-free survival (DFS) benefits from adjuvant atezolizumab versus other PD-L1 expression subgroups (14). Overall, PD-L1 expression levels are positively correlated with ICI therapeutic benefits, with higher expression typically predicting more favorable outcomes (15).

Given this association, precise assessment of PD-L1 expression status prior to formulating neoadjuvant treatment strategies is important. Currently, immunohistochemistry (IHC) is the clinical gold standard for detecting PD-L1 expression. However, this method relies on invasive tissue biopsies and is limited by sampling bias, spatial tumor heterogeneity and high detection costs (16), underscoring a clinical need for a non-invasive, convenient and efficient tool for PD-L1 expression screening.

In recent years, machine learning (ML)-based artificial intelligence has been increasingly integrated into clinical oncology, providing powerful tools for modeling medical data and predicting clinical outcomes, including PD-L1 expression status in lung cancer (17). Compared with conventional imaging modalities, [^18^F]FDG PET/CT not only yields clear anatomical features via CT, but also directly reflects tumor metabolic activity using parameters such as the maximum standardized uptake value (SUVmax). This hypermetabolic profile has been demonstrated to correlate with the tumor immune microenvironment and PD-L1 expression levels (18).

Beyond [^18^F]FDG PET/CT, emerging molecular and immuno-PET agents have further expanded the non-invasive toolkit for evaluating the tumor immune microenvironment. Notably, the first clinical experience with ^68^Ga-FAPI PET/CT demonstrated its feasibility across multiple solid tumors including lung cancer, with tracer uptake strongly correlating with tumor grade (Spearman’s rho = 0.83, P < 0.00001) and altering treatment decisions in 35.5% of cases, though musculoskeletal degenerative changes remained a common diagnostic pitfall (19). A dedicated review further confirmed that FAPI PET/CT achieves superior specificity for lymph node staging compared with [^18^F]FDG PET/CT (94% *vs*. 66%) (20). In parallel, immunoPET employing radiolabeled tracers targeting PD-L1 or CD8^+^ T cells enables direct *in vivo* visualization of immunotherapy targets and immune cell infiltration (21). Despite these advances, the application of such novel PET modalities in resectable NSCLC remains limited, whereas [^18^F]FDG PET/CT, the most widely accessible modality, holds substantial and underexplored potential for predicting PD-L1 high expression in this setting.

Previous studies have demonstrated that ML models constructed from [^18^F]FDG PET/CT or CT radiomic features can effectively predict PD-L1 positivity (TPS ≥ 1%) (22–24). However, these studies rarely stratified NSCLC patients by disease stage, despite significant biological heterogeneity (e.g., in proliferative activity and immune microenvironment) between Stage I-III resectable NSCLC and advanced disease.

To date, systematic research remains scarce on the integration of multiple ML algorithms, [^18^F]FDG PET/CT metabolic parameters and clinicopathological features for the specific prediction of PD-L1 high expression (TPS ≥ 50%) in patients with Stage I-III resectable NSCLC. Furthermore, most existing studies focus on binary PD-L1 positivity prediction or utilize a single ML model, lacking systematic cross-algorithm comparisons to identify the optimal model for clinical translation. In the context of neoadjuvant chemoimmunotherapy (nCIT), pre-treatment precise prediction of PD-L1 high expression holds substantial clinical value for identifying genuine candidates for immunotherapy and avoiding over-treatment and immune-related adverse events.

This study aimed to investigate and comprehensively compare the predictive performance of eight ML models incorporating [^18^F]FDG PET/CT imaging data and clinicopathological features for PD-L1 high expression in patients with Stage IB-III resectable NSCLC. Ultimately, we sought to construct a stable, efficient and clinically applicable predictive nomogram to facilitate personalized treatment decision-making in the neoadjuvant setting for NSCLC.

## 2 Methods

### 2.1 Patient selection

A dual-center retrospective analysis was conducted, continuously recruiting patients diagnosed with operable NSCLC undergoing nCIT. Participants were sourced from Guangdong Provincial People’s Hospital (between January 2019 and December 2023) and the First Affiliated Hospital of Shantou University (between January 2020 and December 2024). Inclusion criteria were: (1)clinical stage IB–IIIB NSCLC, with TNM stage determined by [^18^F]FDG PET/CT imaging in combination with pre-treatment biopsy pathology, according to the 8th edition of the AJCC lung cancer TNM staging system; (2) completion of standard nCIT followed by surgical excision, alongside pathology records; (3) an [^18^F]FDG PET/CT scan performed no more than 14 days prior to starting nCIT, with full diagnostic interpretations; (4) successful PD-L1 evaluation via IHC utilizing the 22C3 clone, yielding adequate tissue and definitive TPS results; and (5) availability of all essential clinical, imaging, and pathological parameters. Criteria for exclusion comprised: (1) concurrent or previous alternate malignancies; (2) inadequate tissue samples for PD-L1 (22C3) assessment or ambiguous TPS evaluations; (3) exposure to prior oncological treatments, including radiation or targeted agents, prior to operative intervention; and (4) concurrent severe organ failures affecting the heart, lungs, liver, or kidneys, which would prevent adherence to the planned therapeutic protocol.

Both institutional review boards approved the study protocol (Guangdong Provincial People’s Hospital: KY2023-351-02; First Affiliated Hospital of Shantou University: B-2025-180), in accordance with the Declaration of Helsinki. Written informed consent was waived due to the retrospective nature of the study, and all data were fully anonymized to protect patient privacy. The dataset was divided by institution: 216 patients from Guangdong Provincial People’s Hospital constituted the training cohort for model development and refinement, while 53 patients from the First Affiliated Hospital of Shantou University served as an independent external validation cohort.

### 2.2 PD-L1 expression assessment

PD-L1 levels were assessed utilizing formalin-fixed, paraffin-embedded (FFPE) materials sourced from pre-treatment biopsies or resected surgical specimens. Staining procedures employed the 22C3 monoclonal antibody (pharmDx), strictly following established clinical protocols and the manufacturer’s guidelines. Two experienced pathologists, blinded to the patients’ clinical histories, imaging findings, and therapeutic responses, independently scored the samples. The primary metric was the TPS, which represents the fraction of viable tumor cells displaying any degree of membrane staining. Based on these scores, patients were classified into three distinct groups: negative expression (< 1%), low expression (1%–49%), and high expression (≥ 50%, the primary endpoint of this study). Any score below 50% was categorized together as “PD-L1 non-high expression,” which merged both the low and negative cohorts. When the initial two pathologists disagreed, a third senior lung pathology expert reviewed the slide to establish a definitive diagnosis via collaborative discussion. Inter-observer reliability was measured using Cohen’s Kappa, with a score of ≥ 0.75 indicating strong concordance. All laboratory steps adhered to strict, standardized quality assurance protocols.

### 2.3 [^18^F]FDG PET/CT acquisition and analysis

PET/CT imaging was performed using either a Siemens Biograph mCT64 (Siemens Healthineers, Erlangen, Germany) or a GE Discovery MI (GE Healthcare, Boston, USA). All patients fasted for at least six hours prior to radiotracer injection, and blood glucose levels were confirmed <150 mg/dL (≈ 8.3 mmol/L). Participants received 0.10–0.15 mCi/kg (≈ 3.7–5.55 MBq/kg) of [^18^F]FDG intravenously. After a 60-minute uptake period, including 500 mL of warm water ingestion, low-dose CT (vertex-to-thigh) and PET scans were acquired. Both centers followed standardized acquisition protocols, including daily phantom quality control, consistent patient preparation, FDG dosage, and uptake times. SUVmax was processed as a binary variable to reduce sensitivity to inter-scanner variability. Data from each center were kept as separate datasets. The primary lesion SUVmax was defined as the principal metabolic indicator. Active lesions were those with FDG uptake exceeding mediastinal blood pool and SUVmax ≥ 2.5. Two nuclear medicine physicians, blinded to clinical outcomes and PD-L1 status, manually delineated regions of interest (ROIs) on fused PET/CT images using syngo.via (Siemens) or AW VolumeShare 7 (GE). Discrepancies were resolved by consensus, with a third senior physician as final arbiter.

### 2.4 Data collection

Pre-treatment baseline characteristics were systematically gathered from hospital databases, pathology archives, and radiology records. Captured clinical and pathological variables comprised: age, sex, body mass index (BMI), tobacco use history (non-smokers *vs*. smokers, encompassing both current and former smokers), histological subtype (adenocarcinoma/squamous cell carcinoma/others), degree of cellular differentiation (well/moderately/poorly differentiated), primary lesion site, alongside clinical T, N, and overall TNM classifications, determined according to AJCC 8th edition guidelines (25). Extracted radiological metrics included the target tumor’s SUVmax and its longest axial dimension on CT (CTlong). The targeted prediction endpoint was the dichotomized PD-L1 status (high expression: TPS ≥ 50%; non-high expression: TPS < 50%). Two independent investigators performed the data harvesting, while a third senior clinician audited the records to resolve inconsistencies.

### 2.5 Feature preprocessing and machine learning model construction

To improve practical clinical utility and minimize outlier effects, the primary tumor’s SUVmax was dichotomized using an optimal diagnostic threshold determined by Youden’s Index derived from the receiver operating characteristic (ROC) curve of the training cohort. Subsequently, a least absolute shrinkage and selection operator (LASSO) framework was applied to the training cohort to compress the high-dimensional variable space. The critical predictors that retained non-zero coefficients were extracted via a 10-fold cross-validation procedure. Of note, this variable selection follows a prediction-optimization framework: multivariable logistic regression was conducted only for assessing independent associations and providing a benchmark for comparison, while the final feature set was determined by a composite evaluation integrating LASSO coefficient stability, AIC improvement, and clinical net benefit on Decision Curve Analysis.

Utilizing the refined feature set, eight distinct classification frameworks were built and contrasted: Logistic Regression (LR), Random Forest (RF), Support Vector Machine (SVM), Decision Tree (DT), K-Nearest Neighbors (KNN), Naive Bayes (NB), Neural Network (NN), and eXtreme Gradient Boosting (XGBoost). To secure optimal hyperparameter settings that resist overfitting and ensure generalizability, every model underwent an internal validation scheme involving 10-fold cross-validation, iterated three times within the training set. Hyperparameter tuning relied on a Grid Search approach, prioritizing the maximization of the area under the ROC curve (AUC).

### 2.6 Dual-validation strategy

A comprehensive two-tier evaluation methodology that combines internal resampling with an independent external validation group was implemented to authenticate the models’ stability and practical adaptability. First, the training dataset was subjected to a 1000-iteration Bootstrap resampling protocol. During each cycle, algorithms were trained on a bootstrap sample and subsequently evaluated against the out-of-bag (OOB) samples. By averaging the results and calculating 95% confidence intervals (95% CI) across all iterations, the optimism bias typically associated with algorithmic overfitting was partially addressed. Finally, the eight trained models derived from the training phase were directly tested on the external validation cohort to evaluate their performance on previously unseen data.

### 2.7 Performance evaluation

The primary measure of predictive performance was the AUC. Because these ML methods yield continuous probability scores, Youden’s Index was applied to the ROC curves of the training cohort to identify the optimal probability cutoff for each individual model. These pre-determined thresholds were then applied to the validation data to construct confusion matrices, enabling the derivation of Sensitivity, Specificity, Accuracy, Precision, and the F1-Score. Within the external cohort, the DeLong method was utilized for pairwise comparisons to identify significant discrepancies in AUC between sophisticated machine learning tools and the standard clinical approach (represented by LR). To limit Type I error inflation caused by multiple hypothesis testing, all derived P-values were adjusted using the Bonferroni correction. Finally, the best-performing architecture underwent further scrutiny via calibration plots and Decision Curve Analysis (DCA) to confirm predictive reliability and quantify tangible clinical net benefits.

### 2.8 Statistical analysis

Data processing and algorithmic programming were entirely executed within R software (version 4.3.2, R Foundation for Statistical Computing, Vienna, Austria). Continuous measurements demonstrating non-normal distributions were summarized as medians alongside their interquartile ranges (IQR), and variations between groups were assessed using the Wilcoxon rank-sum test. Categorical variables were detailed as absolute counts and relative percentages, with group differences evaluated through either the Chi-square test or Fisher’s exact test, as appropriate. All statistical tests were two-tailed, defining an adjusted P-value of < 0.05 as the threshold for statistical significance.

## 3 Results

### 3.1 Baseline characteristics of the study cohorts

A total of 269 patients with NSCLC from two clinical centers were enrolled and divided into a training cohort (n = 216) and an independent external validation cohort (n = 53) based on their institutional origin. In the overall cohort, 79.9% (215/269) were male and 32.0% (86/269) were older than 65 years. Neoadjuvant treatment regimens are detailed in **Supplementary Table 3**. As shown in **Table 1**, demographic characteristics, clinical staging, and imaging parameters demonstrated balanced distributions between the two cohorts (all P > 0.05). To explore clinical and biological differences related to PD-L1 expression, the entire cohort was stratified into a high-expression group (TPS ≥ 50%) and a non-high-expression group (TPS < 50%) for comparative analysis (**Table 2**). The results indicated that high PD-L1 expression was significantly enriched in patients with a history of smoking, the lung squamous cell carcinoma (LUSC) subtype, poor differentiation (Grade 3-4), and advanced T stage (T3-T4) (all P < 0.05). Regarding radiological evaluations, the high-expression group had a larger tumor burden (maximal CT dimension, P = 0.009) and higher metabolic activity (SUVmax, P = 0.007). Conversely, variables including sex, age, BMI, tumor location, and clinical N stage showed no statistical differences between the two groups (all P > 0.05).

**Table 1:** Baseline clinicopathological characteristics of the study cohorts.

Variable	Overall (N = 269)^1^	Dataset		P value^2^
		Training (n = 216)^1^	Validation (n = 53)^1^	
Sex				0.185
Female	54.0 (20.1%)	47.0 (21.8%)	7.0 (13.2%)	
Male	215.0 (79.9%)	169.0 (78.2%)	46.0 (86.8%)	
Age				0.327
>65	86.0 (32.0%)	66.0 (30.6%)	20.0 (37.7%)	
≤65	183.0 (68.0%)	150.0 (69.4%)	33.0 (62.3%)	
Smoking status				0.760
Non-smoker	128.0 (47.6%)	104.0 (48.1%)	24.0 (45.3%)	
Smoker	141.0 (52.4%)	112.0 (51.9%)	29.0 (54.7%)	
BMI				0.123
<25	196.0 (72.9%)	162.0 (75.0%)	34.0 (64.2%)	
≥25	73.0 (27.1%)	54.0 (25.0%)	19.0 (35.8%)	
Histological type				0.506
LUAD	139.0 (51.7%)	115.0 (53.2%)	24.0 (45.3%)	
LUSC	108.0 (40.1%)	83.0 (38.4%)	25.0 (47.2%)	
Others	22.0 (8.2%)	18.0 (8.3%)	4.0 (7.5%)	
N stage				0.956
0	49.0 (18.2%)	38.0 (17.6%)	11.0 (20.8%)	
1	46.0 (17.1%)	37.0 (17.1%)	9.0 (17.0%)	
2	127.0 (47.2%)	103.0 (47.7%)	24.0 (45.3%)	
3	47.0 (17.5%)	38.0 (17.6%)	9.0 (17.0%)	
T stage				0.540
T1-2	135.0 (50.2%)	106.0 (49.1%)	29.0 (54.7%)	
T3-4	134.0 (49.8%)	110.0 (50.9%)	24.0 (45.3%)	
Tumor site				0.931
Left lower lobe	44.0 (16.4%)	36.0 (16.7%)	8.0 (15.1%)	
Left upper lobe	66.0 (24.5%)	53.0 (24.5%)	13.0 (24.5%)	
Right lower lobe	56.0 (20.8%)	43.0 (19.9%)	13.0 (24.5%)	
Right middle lobe	18.0 (6.7%)	14.0 (6.5%)	4.0 (7.5%)	
Right upper lobe	85.0 (31.6%)	70.0 (32.4%)	15.0 (28.3%)	
Clinical TNM stage				>0.999
I - II	64.0 (23.8%)	52.0 (24.1%)	12.0 (22.6%)	
III	205.0 (76.2%)	164.0 (75.9%)	41.0 (77.4%)	
Histological grade				0.757
Grade 1-2	156.0 (58.0%)	124.0 (57.4%)	32.0 (60.4%)	
Grade 3-4	113.0 (42.0%)	92.0 (42.6%)	21.0 (39.6%)	
Location side				0.877
Left	110.0 (40.9%)	89.0 (41.2%)	21.0 (39.6%)	
Right	159.0 (59.1%)	127.0 (58.8%)	32.0 (60.4%)	
SUVmax				>0.999
< 17.6	195.0 (72.5%)	156.0 (72.2%)	39.0 (73.6%)	
≥ 17.6	74.0 (27.5%)	60.0 (27.8%)	14.0 (26.4%)	
PD-L1 expression				0.558
TPS < 50%	218.0 (81.0%)	173.0 (80.1%)	45.0 (84.9%)	
TPS≥50%	51.0 (19.0%)	43.0 (19.9%)	8.0 (15.1%)	
SUVmax (continuous)				0.509
Median (Q1, Q3)	13.4 (8.4, 18.1)	13.7 (8.5, 18.2)	12.3 (8.4, 17.8)	
CT1 (long-axis diameter)				0.665
Median (Q1, Q3)	2.8 (2.1, 3.6)	2.8 (2.1, 3.6)	2.8 (2.5, 3.8)	

SUVmax, maximum standardized uptake value; PD-L1, programmed death-ligand 1; BMI, body mass index; LUAD, lung adenocarcinoma; LUSC, lung squamous cell carcinoma, Others includes lymphoepithelioma-like carcinoma, adenosquamous carcinoma, large cell neuroendocrine carcinoma, high-grade neuroendocrine carcinoma, neuroendocrine carcinoma, neuroendocrine microtumor, and NUT carcinoma, all of which belong to the pulmonary neuroendocrine tumor spectrum or other rare subtypes distinct from adenocarcinoma and squamous cell carcinoma.

**Table 2:** Baseline characteristics stratified by PD-L1 expression status in the overall cohort.

Variable	Overall (N = 269)^1^	PD-L1 expression		P value^2^
		PDL1 high (n = 51)^1^	PDL1 low (n = 218)^1^	
Sex				0.846
Female	54.0 (20.1%)	11.0 (21.6%)	43.0 (19.7%)	
Male	215.0 (79.9%)	40.0 (78.4%)	175.0 (80.3%)	
Age,				0.868
>65	86.0 (32.0%)	17.0 (33.3%)	69.0 (31.7%)	
≤65	183.0 (68.0%)	34.0 (66.7%)	149.0 (68.3%)	
Smoking status				<0.001
Non_smoker	128.0 (47.6%)	13.0 (25.5%)	115.0 (52.8%)	
Smoker	141.0 (52.4%)	38.0 (74.5%)	103.0 (47.2%)	
BMI				0.115
<25	196.0 (72.9%)	42.0 (82.4%)	154.0 (70.6%)	
≥25	73.0 (27.1%)	9.0 (17.6%)	64.0 (29.4%)	
Histological type				<0.001
LUAD	139.0 (51.7%)	18.0 (35.3%)	121.0 (55.5%)	
LUSC	108.0 (40.1%)	32.0 (62.7%)	76.0 (34.9%)	
Others	22.0 (8.2%)	1.0 (2.0%)	21.0 (9.6%)	
N stage				0.501
0	49.0 (18.2%)	13.0 (25.5%)	36.0 (16.5%)	
1	46.0 (17.1%)	8.0 (15.7%)	38.0 (17.4%)	
2	127.0 (47.2%)	23.0 (45.1%)	104.0 (47.7%)	
3	47.0 (17.5%)	7.0 (13.7%)	40.0 (18.3%)	
T stage				0.001
T1-2	135.0 (50.2%)	15.0 (29.4%)	120.0 (55.0%)	
T3-4	134.0 (49.8%)	36.0 (70.6%)	98.0 (45.0%)	
Tumor site				0.495
Left lower lobe	44.0 (16.4%)	5.0 (9.8%)	39.0 (17.9%)	
Left upper lobe	66.0 (24.5%)	12.0 (23.5%)	54.0 (24.8%)	
Right lower lobe	56.0 (20.8%)	10.0 (19.6%)	46.0 (21.1%)	
Right middle lobe	18.0 (6.7%)	5.0 (9.8%)	13.0 (6.0%)	
Right upper lobe	85.0 (31.6%)	19.0 (37.3%)	66.0 (30.3%)	
Clinical TNM stage				0.472
I - II	64.0 (23.8%)	14.0 (27.5%)	50.0 (22.9%)	
III	205.0 (76.2%)	37.0 (72.5%)	168.0 (77.1%)	
Histological grade				<0.001
Grade 1-2	156.0 (58.0%)	14.0 (27.5%)	142.0 (65.1%)	
Grade 3-4	113.0 (42.0%)	37.0 (72.5%)	76.0 (34.9%)	
Location side				0.269
Left	110.0 (40.9%)	17.0 (33.3%)	93.0 (42.7%)	
Right	159.0 (59.1%)	34.0 (66.7%)	125.0 (57.3%)	
SUVmax				<0.001
< 17.6	195.0 (72.5%)	25.0 (49.0%)	170.0 (78.0%)	
≥ 17.6	74.0 (27.5%)	26.0 (51.0%)	48.0 (22.0%)	
CT1 (long-axis diameter)				0.009
Median (Q1, Q3)	2.8 (2.1, 3.6)	3.4 (2.4, 4.0)	2.7 (2.0, 3.4)	
SUVmax (continuous)				0.007
Median (Q1, Q3)	13.3 (8.0, 18.1)	17.6 (10.4, 20.9)	12.6 (7.8, 17.3)	

SUVmax, maximum standardized uptake value; PD-L1, programmed death-ligand 1; BMI, body mass index; LUAD, lung adenocarcinoma; LUSC, lung squamous cell carcinoma; Others, includes lymphoepithelioma-like carcinoma, adenosquamous carcinoma, large cell neuroendocrine carcinoma, high-grade neuroendocrine carcinoma, neuroendocrine carcinoma, neuroendocrine microtumor, and NUT carcinoma (all of which belong to the pulmonary neuroendocrine tumor spectrum or other rare subtypes distinct from adenocarcinoma and squamous cell carcinoma).

In the analysis of PET metabolic features, the median primary tumor SUVmax was higher in the high-expression group compared to the non-high-expression counterpart (17.6 *vs*. 12.6). A three-tier subgroup trend analysis (**Supplementary Figure 1A**) further verified a progressive escalation of SUVmax alongside increasing PD-L1 expression gradients (P = 0.014). *Post-hoc* pairwise comparisons revealed significant statistical disparities between the high-expression group and the other two subgroups, whereas the low-expression and negative groups demonstrated comparable metabolic profiles. The optimal SUVmax cutoff for predicting high PD-L1 expression was established at 17.6 via Youden’s index (**Supplementary Figure 1B**). This dichotomized SUVmax was therefore included as a candidate feature for subsequent model construction.

### 3.2 Feature selection and independent predictors

**Figure 1:**
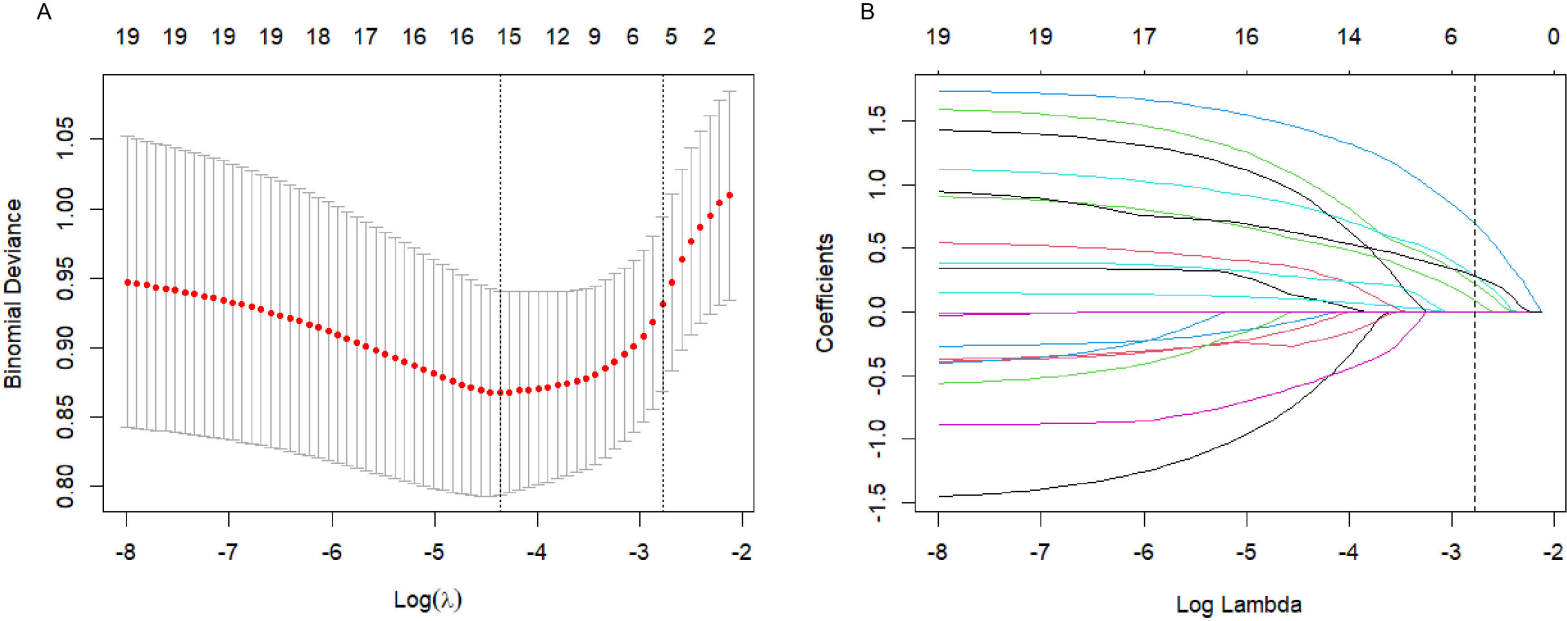
Feature selection utilizing the least absolute shrinkage and selection operator (LASSO) regression. **(A)** Tuning parameter (λ) selection via 10-fold cross-validation. The optimal λ was determined at the minimum binomial deviance (left dashed line) and the 1-standard error (1-SE) criteria (right dashed line). **(B)** LASSO coefficient profiles of the clinical-pathological and metabolic features.

To eliminate multicollinearity among variables and mitigate the risk of overfitting, LASSO regression was applied within the training set to compress baseline features. Utilizing 10-fold cross-validation, the optimal penalty parameter λ was determined under the principle of minimizing binomial deviance. At the 1-SE criteria, the coefficient path of the model illustrated good selection stability (**Figure 1**). Ultimately, five core features were isolated for model construction: smoking history, histological subtype, T stage, tumor differentiation, and dichotomized SUVmax. Both univariate and multivariate logistic regression analyses (**Supplementary Table 1**) demonstrated that a smoking history (OR = 2.483), the LUSC subtype (OR = 2.466), advanced T stage (OR = 2.639), and poor differentiation (OR = 5.922) served as independent predictive factors for high PD-L1 expression (all P < 0.05). Although the independent statistical significance of the dichotomized SUVmax was limited in the multivariate logistic model (OR = 1.92, P = 0.121), it was ultimately retained as a core predictive feature. This decision comprehensively accounted for its stable coefficient within the LASSO algorithm, a lower Akaike Information Criterion (AIC: 178.93 *vs*. 179.43), and enhanced clinical net benefit across a broader range of thresholds (**Supplementary Figure 2**).

### 3.3 Comprehensive comparison of machine learning models

**Figure 2:**
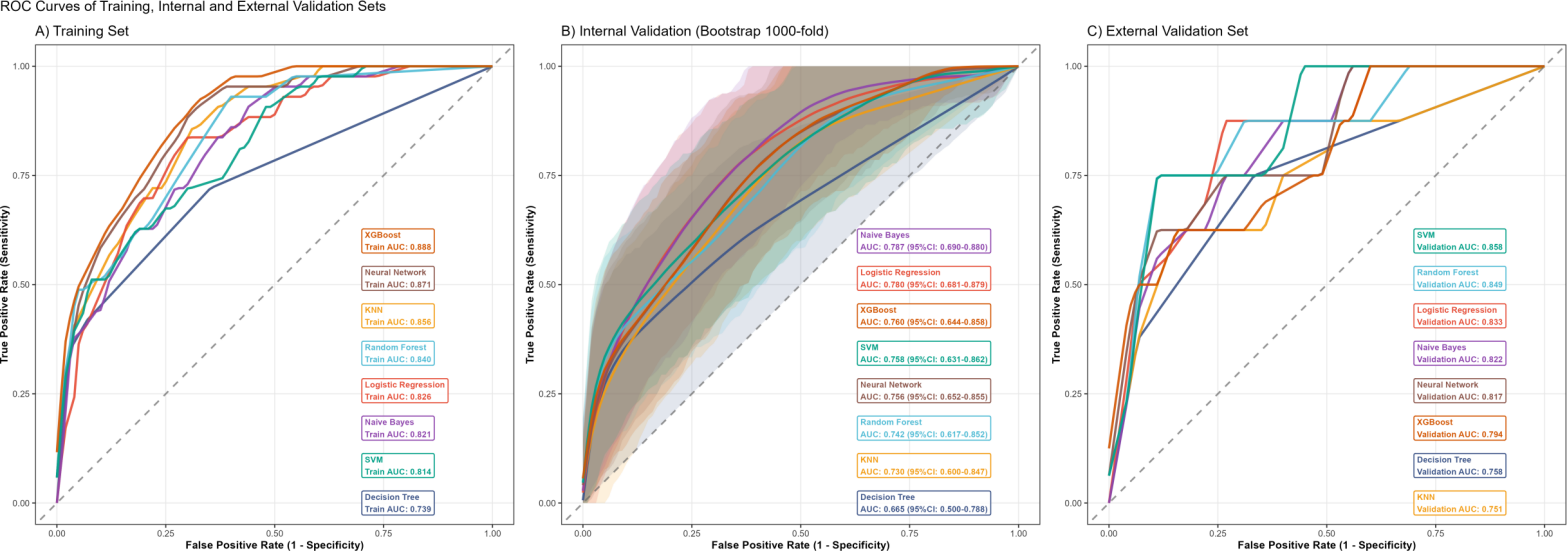
Receiver operating characteristic (ROC) curves of the eight machine learning models across different cohorts. **(A)** Performance in the training set. **(B)** Performance during the 1000-fold Bootstrap internal validation, with shaded areas representing the 95% confidence intervals (CI). **(C)** Generalization performance in the independent external validation set.

**Figure 3:**
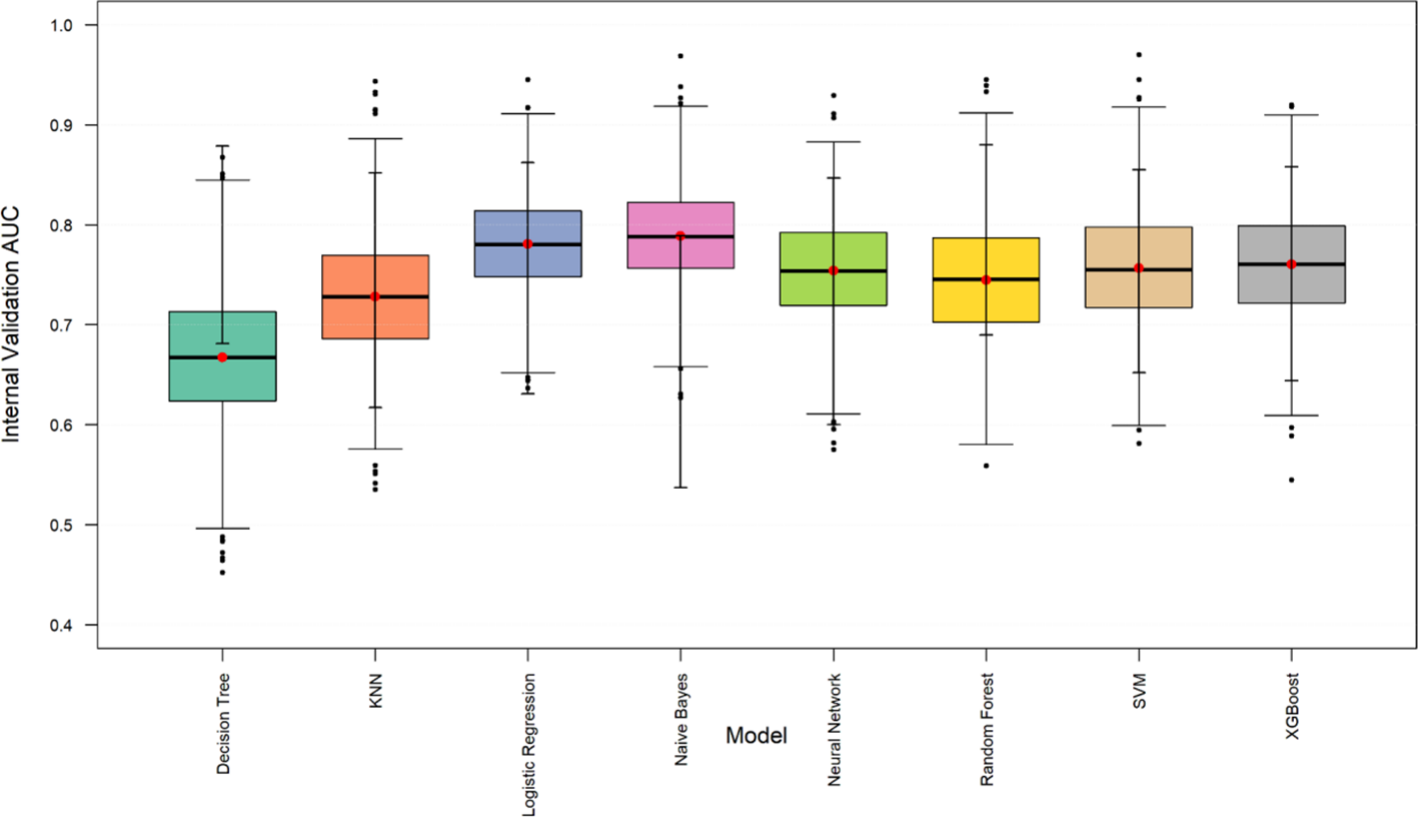
Distribution of the internal validation discriminative performance across eight machine learning algorithms. Boxplots demonstrate the area under the curve (AUC) values derived from 1,000 Bootstrap resamples for each model. The central line indicates the median AUC, the red dot represents the mean AUC, and the box spans the interquartile range (IQR). KNN, K-Nearest Neighbors; SVM, Support Vector Machine; XGBoost, eXtreme Gradient Boosting.

**Figure 4:**
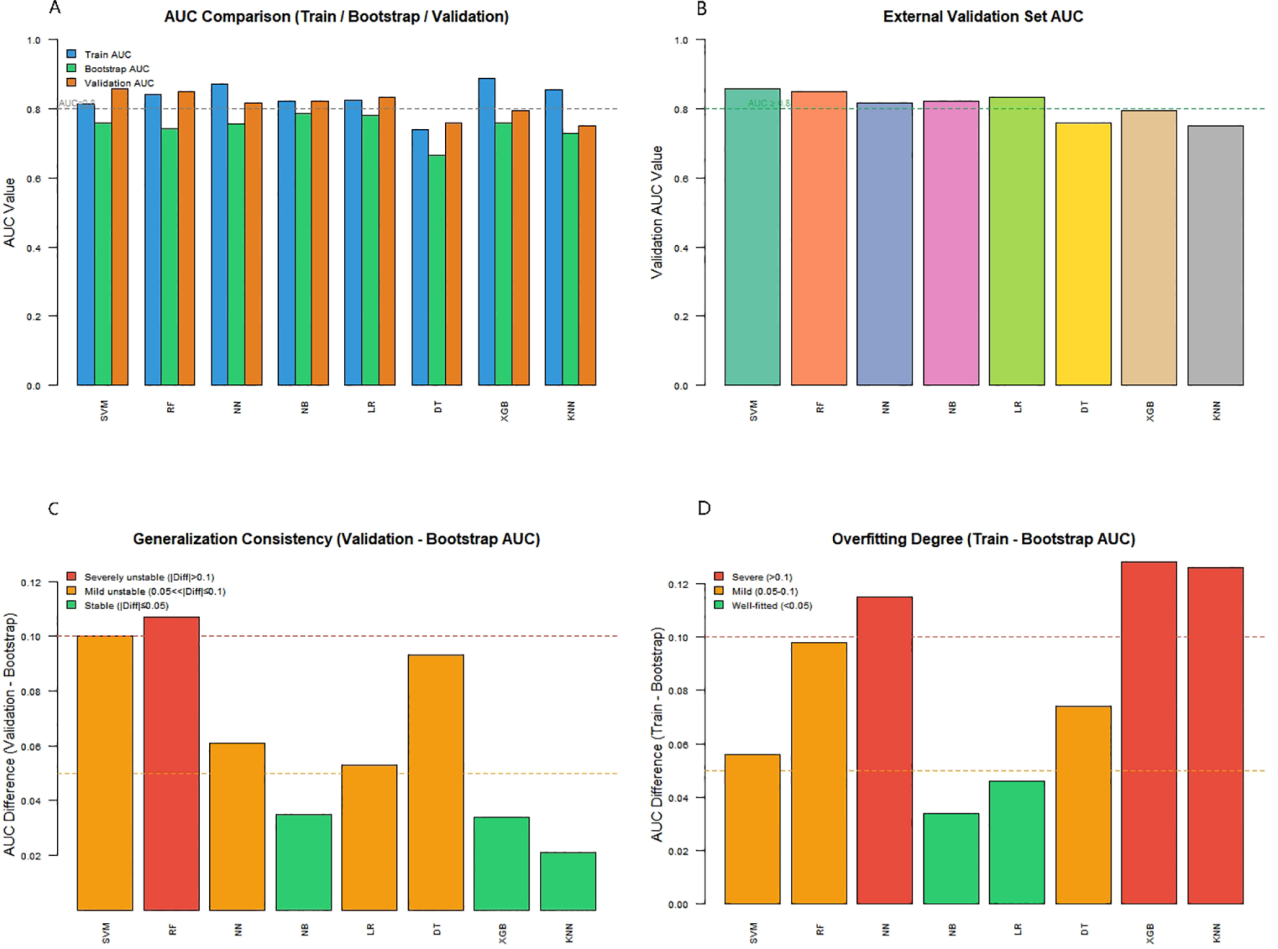
Comprehensive evaluation of machine learning models for generalization, stability, and overfitting risks. **(A)** Bar chart showing the AUC values of eight models in the training set, 1000-fold Bootstrap internal validation, and independent external validation; **(B)** Bar chart ranking the external validation AUC values of eight models, with the highest AUC values observed for SVM, RF, LR, and NB; **(C)** Bar chart evaluating generalization consistency, quantified as the AUC difference (external validation AUC minus Bootstrap AUC); positive values indicate superior cross-cohort stability, while negative values reflect performance degradation; **(D)** Bar chart assessing overfitting degree, calculated as the AUC difference (training AUC minus Bootstrap AUC); larger values indicate more severe overfitting of complex models on training data.

**Figure 5:**
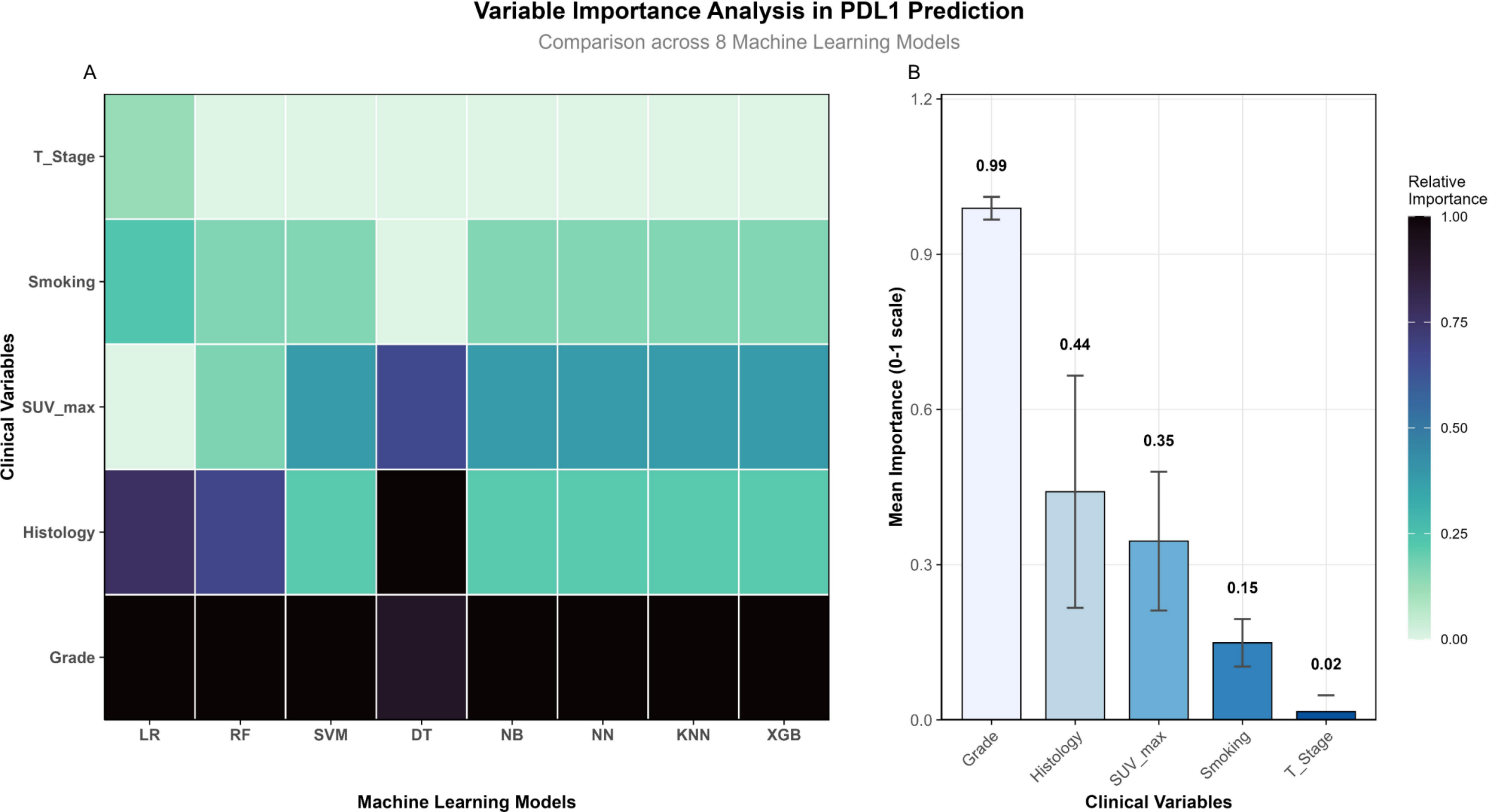
Variable importance analysis for PD-L1 high expression prediction across eight machine learning models. **(A)** Heatmap of relative variable importance across models (darker = higher predictive weight), showing histological grade (Grade) as the dominant predictor. **(B)** Bar chart of mean relative importance (0–1 scale, error bars = SD).

Following feature selection, eight machine learning algorithms were trained using the identified core predictors. The discriminative performance of these models, optimized via Youden’s index, is detailed in **Table 3**. The corresponding ROC curves illustrating their fitting efficacy in the training set are presented in **Figure 2A**. During the 1000-iteration Bootstrap internal validation (**Figures 2B**, **3**), divergent performance metrics emerged among the models. Specifically, NB and LR displayed good internal predictive efficacy and stability, yielding average Bootstrap AUCs of 0.787 (95% CI: 0.690-0.880) and 0.780 (95% CI: 0.681-0.879), respectively. Within the independent external validation cohort (**Figure 2C**), over half of the algorithms achieved favorable predictive performance (AUC > 0.80). SVM (AUC = 0.858, 95% CI: 0.715-0.862), RF (AUC = 0.849, 95% CI: 0.687-0.864), LR (AUC = 0.833, 95% CI: 0.681-0.839), and NB (AUC = 0.822, 95% CI: 0.662-0.826) ranked as the top four models. Evaluations of overfitting (Training AUC minus Bootstrap AUC) and generalization consistency (External Validation AUC minus Bootstrap AUC) revealed that despite XGBoost and NN achieving the highest AUCs during training (0.888 and 0.871, respectively), they suffered from severe overfitting (both differences > 0.10) (**Figure 4**). Regarding cross-cohort generalization, SVM and RF exhibited significant performance fluctuations between internal and external validations, whereas the generalization consistency of the LR and NB models remained highly stable. Furthermore, a global variable importance analysis was conducted across all eight predictive models (**Figure 5**). Normalized results indicated that histological grade and subtype provided the highest information gain in the decision-making logic of most algorithms. The primary tumor SUVmax consistently secured the third position in overall importance (average relative importance score: 0.35), surpassing both smoking history and T stage.

**Table 3:** Comprehensive predictive performance metrics of the eight machine learning models.

Model	Optimal threshold	Training AUC	Bootstrap AUC	Validation AUC	Accuracy	Sensitivity	Specificity	Precision	F1 score
SVM (Support Vector Machine)	0.200	0.814	0.758	0.858	0.887	0.625	0.933	0.625	0.625
RF (Random Forest)	0.100	0.840	0.742	0.849	0.906	0.750	0.933	0.667	0.706
LR (Logistic Regression)	0.200	0.826	0.780	0.833	0.717	0.875	0.689	0.333	0.483
NB (Naive Bayes)	0.100	0.821	0.787	0.822	0.679	0.875	0.644	0.304	0.452
NN (Neural Network)	0.300	0.871	0.756	0.817	0.774	0.750	0.778	0.375	0.500
XGB (Extreme Gradient Boosting)	0.300	0.888	0.760	0.794	0.811	0.625	0.844	0.417	0.500
DT (Decision Tree)	0.250	0.739	0.665	0.758	0.868	0.500	0.933	0.571	0.533
KNN (K-Nearest Neighbors)	0.350	0.856	0.730	0.751	0.868	0.500	0.933	0.571	0.533

The optimal probability threshold for each model was derived from the training set using Youden’s index. Accuracy, Sensitivity, Specificity, Precision, and F1 Score are reported for the external validation cohort based on that threshold.

### 3.4 Determination of the final clinical prediction model

To ascertain whether the superior AUCs of the top models (SVM, RF, NB) in both the bootstrap internal and external validation sets represented a true statistical advantage over LR, the DeLong test was used for pairwise comparisons, with the Bonferroni correction applied to control the false-positive risk (**Supplementary Table 2**).

Throughout the 1000-fold Bootstrap internal validation, the mean AUC discrepancies between NB, RF, SVM, and LR were marginal (differences of 0.006, -0.039, and -0.023, respectively), lacking any statistical significance (P values of 0.920, 0.634, and 0.838, respectively). In the independent external validation cohort, although the absolute point estimates for SVM (AUC = 0.858) and RF (AUC = 0.849) slightly exceeded that of LR (AUC = 0.833), their 95% confidence intervals overlapped substantially. Furthermore, their AUC differences relative to LR were merely 0.025 and 0.016, corresponding to raw P values of 0.743 and 0.629. Following Bonferroni adjustment, all corrected P values for these pairwise comparisons reached 1.000. Grounded in the suggested non-inferiority of the LR model and the principle of model parsimony, LR was ultimately selected as the core architecture for predicting high PD-L1 expression. In addition, Subgroup analysis across histological subtypes (LUAD *vs*. LUSC) showed that the LR model maintained generally stable discriminative performance, as detailed in **Supplementary Figure 3**. The performance metrics of the final LR model at different probability thresholds (0.2 and 0.5) are summarized in **Supplementary Table 4**.

### 3.5 Nomogram construction and clinical utility validation

**Figure 6:**
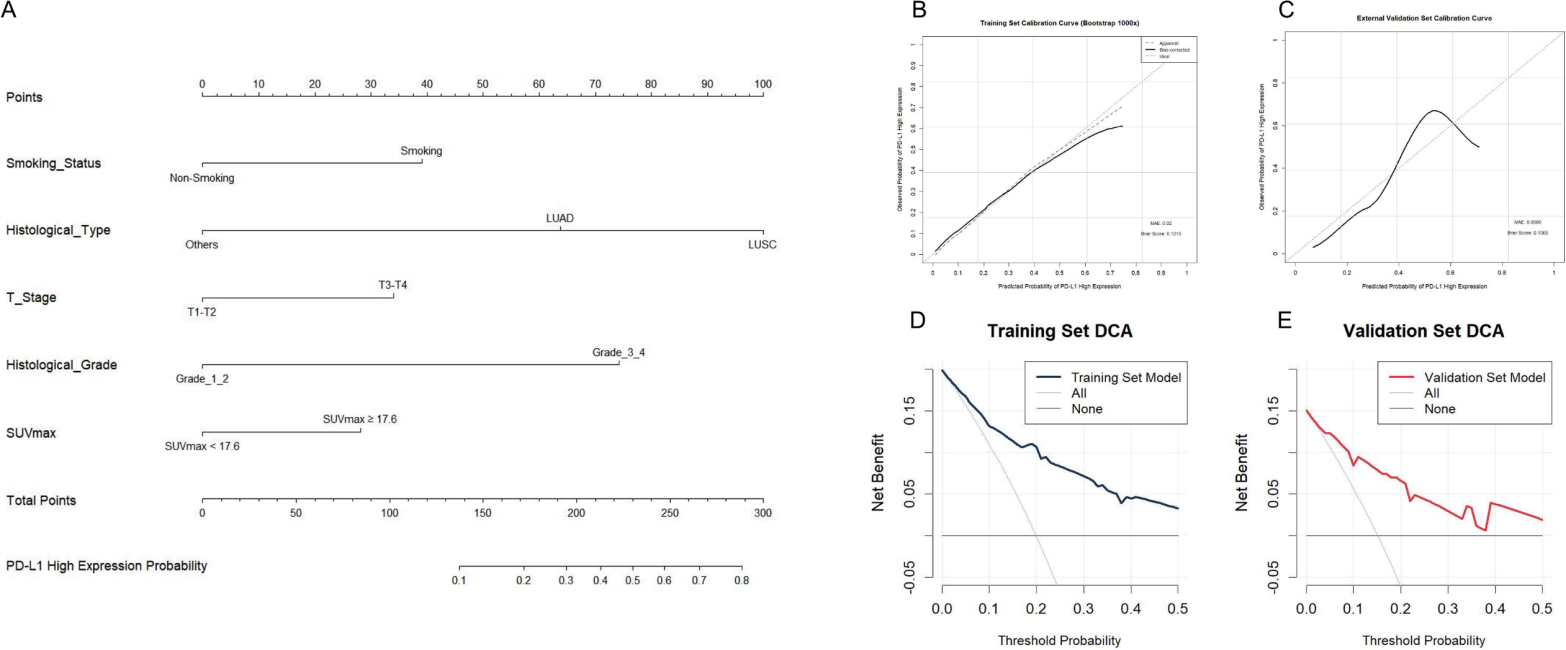
Nomogram development and validation for PD-L1 high expression prediction. **(A)** Nomogram based on five core predictors. **(B, C)** Calibration curves for training **(B)** and external validation **(C)** sets. **(D, E)** DCA curves showing clinical net benefit across a range of thresholds.

Utilizing the LR architecture alongside the five pivotal predictors, a non-invasive nomogram was constructed for personalized prediction of high PD-L1 expression (**Figure 6A**). The corresponding logistic regression equation is: logit(p) = −5.4636+ 0.9290 × (Smoking) + 1.5141 × (LUAD) + 2.3755 × (LUSC) + 0.8088 × (T3−T4) + 1.7638 × (Grade 3−4) + 0.6682 × (SUVmax ≥ 17.6),

Reference categories: Non−Smoking, Others (histology), T1−T2, Grade1-2, SUVmax <17.6

This tool assigns specific weights to each feature, allowing the accumulated total points to directly map the estimated probability of a patient presenting with high PD-L1 expression. Calibration analytics during the 1000-iteration Bootstrap internal validation showed that the model’s predicted probability curve generally aligned with the ideal diagonal. Similarly, the calibration curve in the independent external validation set exhibited reasonable concordance (**Figure 6**). Decision curve analysis (DCA) (**Figures 6D, E**) showed that deploying this nomogram for screening guidance yielded a higher clinical net benefit than either the “treat all” or “treat none” baseline strategies across the evaluated threshold probability ranges, in both training and external validation sets.

## 4 Discussion

In the era of nCIT for resectable NSCLC, the precise identification of patients most likely to derive therapeutic benefit is an important prerequisite for tailored treatment planning. Existing evidence indicates that nCIT improves pathological complete response (pCR) rates and event-free survival (EFS), with these outcomes frequently associated with PD-L1 expression levels (26). Furthermore, the Society of Thoracic Surgeons (STS) consensus emphasizes that high PD-L1 expression correlates with enhanced efficacy following nCIT, supporting routine PD-L1 screening prior to neoadjuvant interventions (27). However, conventional invasive biopsies are often limited by inadequate tissue yield and spatial heterogeneity, meaning that a small biopsy specimen may not fully represent the actual PD-L1 expression status of the entire tumor. Consequently, these factors may lead to underestimation or misclassification of PD-L1 status (16), highlighting a need for non-invasive, accessible screening modalities. To address this clinical challenge, our dual-center retrospective study integrated standard clinicopathological variables with primary tumor SUVmax derived from [^18^F]FDG PET/CT. We systematically evaluated the predictive performance of eight machine learning algorithms, including LR, RF, SVM, DT, KNN, NB, NN, and XGBoost, for identifying high PD-L1 expression. Our analysis identified smoking history, histological subtype, T stage, tumor differentiation grade, and primary tumor SUVmax as key predictors. Through rigorous 1000-iteration Bootstrap internal resampling, independent external validation, and DeLong testing with Bonferroni correction, we observed that the clinically interpretable LR model achieved discriminative performance comparable to more complex ‘black-box’ algorithms, while demonstrating generalization stability across the cross-center external cohort. Based on this architecture, we developed a quantitative nomogram. Our results suggest that this non-invasive approach may serve as a potential auxiliary tool for PD-L1 assessment in the neoadjuvant setting, offering a supplementary perspective for individualized clinical decision-making.

The predictive performance of our model is supported by established biological mechanisms and clinical rationales underlying its core variables. Foremost, we identified primary tumor SUVmax as a meaningful predictor. Although its independent statistical significance was marginal in multivariate logistic regression (P = 0.121), its inclusion was justified by stable coefficients during LASSO compression, a lower model Akaike Information Criterion (AIC: 178.93 *vs*. 179.43), and improved clinical net benefit. From a clinicopathological perspective, this marginal P-value is likely attributable to the biological collinearity between SUVmax, tumor differentiation, and T stage. Specifically, NSCLC lesions with greater tumor burden (advanced T stage) and higher histological grade (Grade 3-4) typically exhibit increased metabolic activity, an observation consistent with findings by Blumenthaler et al. (28). Thus, rather than representing redundant statistical noise, this collinearity reflects a multidimensional characterization of the malignant phenotype. At the molecular level, high PD-L1 expression often coincides with microenvironmental hypoxia and significant upregulation of hypoxia-inducible factor-1 (HIF-1) (29). HIF-1 not only stimulates the transcription of immune checkpoint molecules but concurrently upregulates glucose transporter-1 (GLUT-1) expression, thereby drastically accelerating [^18^F]FDG avidity (30). This metabolism-immunity crosstalk provides a biological basis for the observed coupling between high PD-L1 status and intense glycolytic activity, corroborating evidence from Hu et al., Ishimura et al., and Takada et al. regarding the positive association between SUVmax and PD-L1 intensity (31–33). Notably, Wang et al. reported that in a stage IIIB-IV (generally unresectable) NSCLC cohort, an SUVmax cutoff >17.5 predicted PD-L1 strong positivity (TPS ≥50%) with an accuracy of 84.8%, which aligns well with the cutoff (17.6) used in our study (34). Moreover, our cohort exhibited a stepwise escalation of SUVmax alongside increasing PD-L1 expression gradients, suggesting that PET metabolic parameters may serve as a macroscopic functional window into the immune microenvironment. Nevertheless, the lack of significant metabolic divergence between the low−expression and negative subgroups in our cohort aligns with the findings of Monaco et al. (35), highlighting the predictive limitations of isolated [^18^F]FDG PET/CT parameters and supporting the potential value of combining multiple features.

Beyond metabolic indicators, smoking history was identified as a clinical predictor (OR: 2.483). This finding is consistent with established immunological pathways in which carcinogens, such as polycyclic aromatic hydrocarbons from tobacco combustion, activate the aryl hydrocarbon receptor (AhR) cascade. This activation has been reported to induce compensatory PD-L1 overexpression, potentially facilitating immune evasion and malignant progression (36). Consequently, smoking may serve as a baseline surrogate for assessing potential immune activation. Furthermore, the predictive weights assigned to tumor differentiation, histological subtype, and T stage reflect the influence of intratumoral heterogeneity on the immune microenvironment. Our observation that poorly differentiated (Grade 3-4) lesions and lung squamous cell carcinoma (LUSC) more frequently exhibit high PD-L1 expression is consistent with reports by Hwang et al. (37). Similarly, studies by Pan Y et al. (38) and Zheng et al. (39)linked elevated PD-L1 status with LUSC, larger tumor volumes (advanced T stage), and higher histological grading. The variable importance analysis, integrating multiple algorithms, identified histological grade as a leading predictor, supporting the value of traditional morphological features as indicators.

In addition to these biological predictors, our comparison of eight machine learning algorithms provides preliminary insight into their application in moderately sized real-world cohorts. We observed that complex algorithms were prone to overfitting when sample sizes were limited. Although XGBoost and NN showed good fitting performance during training (both AUCs > 0.87), their performance declined substantially during 1000-fold bootstrap validation (AUC drops > 0.10). This suggests that deep learning and complex tree structures may overfit local data noise rather than capture generalizable biological patterns in smaller datasets, reducing their validation stability. In the independent external validation set, although SVM and RF achieved the highest absolute AUCs (0.858 and 0.849, respectively), DeLong testing with Bonferroni correction indicated that their AUC differences relative to the LR model were not statistically significant (all adjusted P = 1.000). Thus, in this specific context, the more complex ‘black-box’ models did not demonstrate a statistically significant advantage over the simpler LR model.

In complex medical decision-making, model transparency, interpretability, and cross-cohort consistency may outweigh the pursuit of marginal accuracy limits. Adhering to the principle of model parsimony, we selected the simple LR framework as our core architecture. The LR approach maintained smooth performance transitions across cohorts, demonstrating good cross-center stability. Its linear weight mechanism translates easily into an intuitive nomogram, thereby reducing the interpretability barriers inherent to ‘black-box’ algorithms and potentially improving the feasibility and safety of real-world clinical deployment. Clinically, this nomogram may provide some reference value for treatment decisions in resectable NSCLC without requiring additional invasive procedures or substantial economic costs. By fusing macroscopic imaging with microscopic pathology, it may help circumvent the sampling limitations of localized biopsies. The calibration curves indicated reasonable agreement between predicted and observed risks, and DCA suggested potential net benefit of using the nomogram for treatment decisions. Therefore, this non-invasive tool may assist clinicians in developing individualized neoadjuvant immunotherapy regimens. In routine clinical practice, prior to acquiring tissue-based PD-L1 immunohistochemical results, clinicians can perform risk assessment using nomogram. The individualized probability of high PD-L1 expression is obtained by summing the scores of five predictors (histological type, primary tumor SUVmax, T stage, tumor differentiation grade, and smoking history) and matching the total score with the corresponding probability scale. All five predictors are readily obtainable from routine clinical examinations without additional cost or complex post−processing. This predictive model may provide a useful reference for pathological PD-L1 testing results and help address the limitations of preoperative pathological evaluation when biopsy samples are inadequate to define PD-L1 expression status. It may serve as a useful supplement to histopathological evidence and aid clinical decision-making.

Nevertheless, several limitations warrant acknowledgment. Although we performed a dual-center study with external validation, the external cohort was small, and retrospective biases are inherent; thus, prospective confirmation with a larger sample size is required. Secondly, our PET/CT analysis was limited to dichotomized SUVmax, without radiomic features, volumetric parameters (MTV/TLG), or formal cross-center SUV harmonization (e.g., phantom calibration or liver normalization), although standardized acquisition protocols and dichotomization were applied to mitigate scanner variability. Thirdly, pre−treatment biopsies may not capture the full histopathologic heterogeneity of resected tumors. Furthermore, the exclusion of M+ patients limits generalizability to advanced disease. Additionally, LASSO-based feature selection and the determination of the optimal SUVmax cutoff via Youden’s Index on the training cohort may introduce optimism in performance estimates. Moreover, a small subset of external validation patients (8/53, 15.1%) used post−surgical specimens for PD−L1 assessment due to insufficient baseline biopsy tissue, though evidence suggests neoadjuvant therapy does not systematically alter PD−L1 expression. Lastly, PD−L1 was evaluated predominantly with the 22C3 clone; generalizability to other assays (28−8, SP263) requires further validation.

## 5 Conclusion

In conclusion, this study indicates an association between pretreatment primary tumor SUVmax and high PD-L1 expression. By integrating five core features, we comparatively evaluated eight machine learning models and developed an LR-based nomogram. This nomogram holds potential clinical value for identifying patients with PD-L1 high expression (TPS ≥ 50%), particularly when preoperative biopsy tissue is insufficient or non-diagnostic. Prospective validation is still required prior to clinical implementation.

## Data Availability

The raw data supporting the conclusions of this article will be made available by the authors, without undue reservation.

## Supplementary material

Supplementary Table 1 Univariate and multivariate logistic regression analyses for predicting high PD-L1 expression. OR, odds ratio; CI, confidence interval; P-value, statistical significance; BMI, body mass index; LUAD, lung adenocarcinoma; LUSC, lung squamous cell carcinoma; Others, includes lymphoepithelioma-like carcinoma, adenosquamous carcinoma, large cell neuroendocrine carcinoma, high-grade neuroendocrine carcinoma, neuroendocrine carcinoma, neuroendocrine microtumor, and NUT carcinoma (all of which belong to the pulmonary neuroendocrine tumor spectrum or other rare subtypes distinct from adenocarcinoma and squamous cell carcinoma); TPS, tumor proportion score; PD-L1, programmed death-ligand 1; CT1, long-axis diameter of primary tumor.

Supplementary Table 2 Pairwise comparison of the top-performing machine learning models versus logistic regression (LR) using the DeLong test.

Supplementary Table 3 Distributions of neoadjuvant therapeutic regimens in the overall cohort.

Supplementary Table 4 Performance of the LR-based nomogram at different probability thresholds.

Supplementary Figure 1 Association between primary tumor SUVmax and PD-L1 expression levels. **(A)** Boxplots displaying the progressive escalation of SUVmax across non-expression, low-expression, and high-expression PD-L1 groups. Statistical disparities were evaluated using the Kruskal-Wallis test with *post-hoc* pairwise comparisons. **(B)** The ROC curve identifying the optimal primary tumor SUVmax cutoff value (17.6) for discriminating high PD-L1 expression, determined via Youden’s Index.

Supplementary Figure 2 Decision curve analysis (DCA) demonstrating the incremental clinical net benefit of incorporating SUVmax into the predictive framework. The model incorporating SUVmax (red line) provides a superior net benefit compared to the model lacking SUVmax (blue line) across the most clinically relevant high-risk threshold probabilities.

Supplementary Figure 3 Subgroup analysis of the LR model across histological subtypes. AUC values for overall, LUAD, and LUSC subgroups in the training and external validation cohorts.
