# Supplementary material for: Comparative evaluation of machine learning models for predicting PD-L1 high expression in resectable NSCLC: a dual-center study integrating [18F]FDG PET/CT and clinicopathological features
Source: Front Immunol. 2026 Jun 15;17:1834643. doi: 10.3389/fimmu.2026.1834643 (PMC13311110; doi:10.3389/fimmu.2026.1834643)
Supplement: Supplementary Table 1 — Univariate and multivariate logistic regression analyses for predicting high PD-L1 expression. OR, odds ratio; CI, confidence interval; P-value, statistical significance; BMI, body mass index; LUAD, lung adenocarcinoma; LUSC, lung squamous cell carcinoma; Others, includes lymphoepithelioma-like carcinoma, adenosquamous carcinoma, large cell neuroendocrine carcinoma, high-grade neuroendocrine carcinoma, neuroendocrine carcinoma, neuroendocrine microtumor, and NUT carcinoma (all of which belong to the pulmonary neuroendocrine tumor spectrum or other rare subtypes distinct from adenocarcinoma and squamous cell carcinoma); TPS, tumor proportion score; PD-L1, programmed death-ligand 1; CT1, long-axis diameter of primary tumor. [file Table1.docx]

**Supplementary Material**

Supplementary Table S1. Univariate and Multivariate Logistic Regression Analyses for Predicting High PD-L1 Expression

|  |  | Univariable Analysis | | Multivariable Analysis | |
| --- | --- | --- | --- | --- | --- |
| Variable | Level | OR (95%CI) | P.value | OR (95%CI) | P.value |
| Sex | Female | - | - |  |  |
|  | male | 1.063 (0.485 - 2.531) | 0.883 |  |  |
| Age | Age>65 | - | - |  |  |
|  | Age≤65 | 0.780 (0.389 - 1.612) | 0.492 |  |  |
| Smoking | No | - | - |  |  |
|  | Yes | 3.382 (1.645 - 7.424) | 0.001 | 2.483 (1.078 - 6.014) | 0.037 |
| BMI | <25 | - | - |  |  |
|  | ≥25 | 0.753 (0.319 - 1.635) | 0.492 |  |  |
| Histological | LUAD | - | - |  |  |
|  | LUSC | 2.822 (1.411 - 5.798) | 0.004 | 2.466 (1.036 - 6.047) | 0.044 |
|  | Others | 0.364 (0.020 - 1.974) | 0.342 | 0.220 (0.011 - 1.399) | 0.178 |
| Clinical N stage | cN0 | - | - |  |  |
|  | cN1 | 0.624 (0.188 - 1.946) | 0.421 |  |  |
|  | cN2 | 0.926 (0.393 - 2.323) | 0.865 |  |  |
|  | cN3 | 0.488 (0.137 - 1.580) | 0.242 |  |  |
| Clinical T stage | I-II | - | - |  |  |
|  | III-IV | 3.074 (1.514 - 6.597) | 0.003 | 2.639 (1.083 - 6.827) | 0.037 |
| Tumor site | Left lower | - | - |  |  |
|  | Left upper | 1.636 (0.486 - 6.469) | 0.445 |  |  |
|  | Right lower | 1.556 (0.429 - 6.397) | 0.511 |  |  |
|  | Right middle | 4.444 (0.986 - 21.558) | 0.053 |  |  |
|  | Right upper | 2.769 (0.933 - 10.238) | 0.088 |  |  |
| Clinical TNM stage | I - II | - | - |  |  |
|  | III | 1.058 (0.494 - 2.427) | 0.888 |  |  |
| Histological_Grade | Grade 1-2 | - | - |  |  |
|  | Grade 3-4 | 4.743 (2.325 - 10.235) | < 0.001 | 5.922 (2.630 - 14.227) | < 0.001 |
| location_side | Left | - | - |  |  |
|  | Right | 1.808 (0.899 - 3.808) | 0.105 |  |  |
| SUVmax | <17.6 | - | - |  |  |
|  | ≥ 17.6 | 3.722 (1.854 - 7.534) | < 0.001 | 1.924 (0.832 - 4.395) | 0.121 |
| CT1_long |  | 1.335 (1.066 - 1.685) | 0.013 |  |  |
| SUVmax (continuous) |  | 1.046 (1.001 - 1.094) | 0.044 |  |  |
| Age |  | 0.998 (0.962 - 1.036) | 0.909 |  |  |

**Note: OR, odds ratio; CI, confidence interval; P-value, statistical significance; BMI, body mass index; LUAD, lung adenocarcinoma; LUSC, lung squamous cell carcinoma; Others, includes lymphoepithelioma-like carcinoma, adenosquamous carcinoma, large cell neuroendocrine carcinoma, high-grade neuroendocrine carcinoma, neuroendocrine carcinoma, neuroendocrine microtumor, and NUT carcinoma (all of which belong to the pulmonary neuroendocrine tumor spectrum or other rare subtypes distinct from adenocarcinoma and squamous cell carcinoma); TPS, tumor proportion score; PD-L1, programmed death-ligand 1; CT1, long-axis diameter of primary tumor.**

**Supplementary Table S2. Pairwise Comparison of the Top-Performing Machine Learning Models versus Logistic Regression (LR) using the DeLong Test**

| Data | Internal (Bootstrap) |  |  |  |  | External Validation |  |  |  |  |  |
| --- | --- | --- | --- | --- | --- | --- | --- | --- | --- | --- | --- |
|  | LR mean AUC | Model mean AUC | Mean AUC Difference | 95% CI | P Value | LR AUC | Model AUC | AUC Diff | 95% CI | P Value | P (Bonferroni) |
| NB | 0.78 | 0.787 | 0.006 | [-0.135, 0.14] | 0.92 | 0.833 | 0.822 | -0.011 | [-0.034, 0.056] | 0.6272 | 1 |
| RF | 0.78 | 0.742 | -0.039 | [-0.194, 0.107] | 0.634 | 0.833 | 0.849 | 0.016 | [-0.077, 0.047] | 0.6293 | 1 |
| SVM | 0.78 | 0.758 | -0.023 | [-0.176, 0.114] | 0.838 | 0.833 | 0.858 | 0.025 | [-0.174, 0.124] | 0.743 | 1 |
|  | | | | | | | | | | |  |
| Notes: Internal validation = Bootstrap method; External validation = DeLong test (two-sided, α = 0.05); AUC Diff = Model AUC − LR AUC; P < 0.05 was considered statistically significant; LR, logistic regression; NB, naive Bayes; RF, random forest; SVM, support vector machine; AUC, area under the receiver operating characteristic curve; CI, confidence interval. | | | | | | | | | | |  |

**Supplementary Table S3：Distributions of neoadjuvant therapeutic regimens in the Overall Cohort**

| Treatment category | Chemotherapeutic agent | Platinum agent | Immune checkpoint inhibitor | Number of patients |
| --- | --- | --- | --- | --- |
| Taxane-based regimen | Taxane | Carboplatin | Nivolumab | 95 |
| Taxane-based regimen | Taxane | Carboplatin | Tislelizumab | 59 |
| Taxane-based regimen | Taxane | Carboplatin | Sintilimab | 34 |
| Taxane-based regimen | Taxane | Carboplatin | Pembrolizumab | 12 |
| Taxane-based regimen | Taxane | Carboplatin | Camrelizumab | 5 |
| Taxane-based regimen | Taxane | Carboplatin | Toripalimab | 5 |
| Taxane-based regimen | Taxane | Carboplatin | Durvalumab | 3 |
| Pemetrexed-based regimen | Pemetrexed | Carboplatin | Tislelizumab | 19 |
| Pemetrexed-based regimen | Pemetrexed | Carboplatin | Pembrolizumab | 14 |
| Pemetrexed-based regimen | Pemetrexed | Carboplatin | Sintilimab | 11 |
| Pemetrexed-based regimen | Pemetrexed | Carboplatin | Camrelizumab | 6 |
| Pemetrexed-based regimen | Pemetrexed | Cisplatin | Tislelizumab | 3 |
| Pemetrexed-based regimen | Pemetrexed | Carboplatin | Toripalimab | 3 |
| Note: Taxane agents include paclitaxel and albumin-bound paclitaxel. All regimens were administered as preoperative neoadjuvant therapy for 2–4 cycles (21 days/cycle). | | | | |

.

**Supplementary Table S4. Performance of the LR‑based nomogram at different probability thresholds**

| Threshold | Cohort | PPV | NPV | Sensitivity | Specificity | Accuracy | F1 Score |
| --- | --- | --- | --- | --- | --- | --- | --- |
| 0.2 | Training | 0.409 | 0.945 | 0.837 | 0.699 | 0.727 | 0.550 |
| 0.2 | Validation | 0.333 | 0.969 | 0.875 | 0.689 | 0.717 | 0.483 |
| 0.5 | Training | 0.630 | 0.862 | 0.395 | 0.942 | 0.833 | 0.486 |
| 0.5 | Validation | 0.571 | 0.913 | 0.500 | 0.933 | 0.868 | 0.533 |

**A B**

**
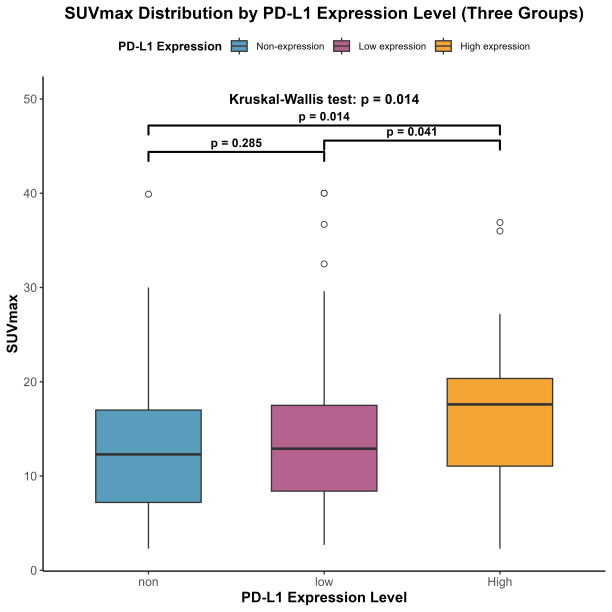

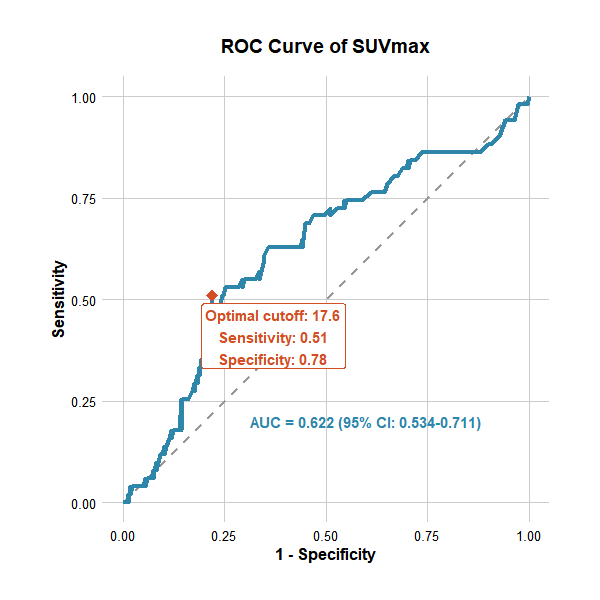
**

**Supplementary Figure S1. Association between primary tumor SUVmax and PD-L1 expression levels. (A) Boxplots displaying the progressive escalation of SUVmax across non-expression, low-expression, and high-expression PD-L1 groups. Statistical disparities were evaluated using the Kruskal-Wallis test with post-hoc pairwise comparisons. (B) The ROC curve identifying the optimal primary tumor SUVmax cutoff value (17.6) for discriminating high PD-L1 expression, determined via Youden's Index.**

**
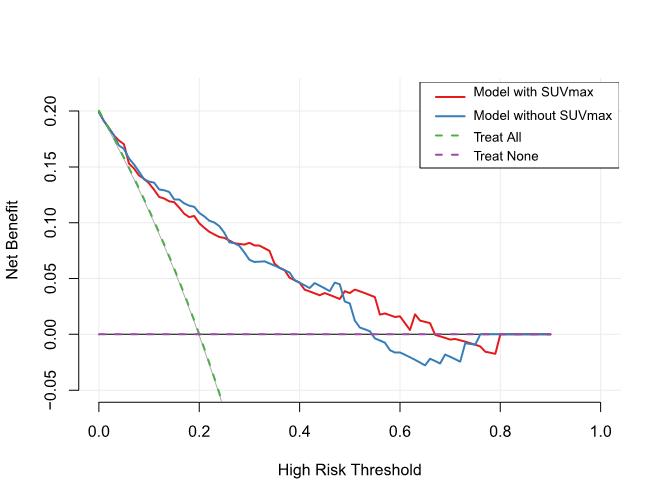
**

**Supplementary Figure S2. Decision curve analysis (DCA) demonstrating the incremental clinical net benefit of incorporating SUVmax into the predictive framework. The model incorporating SUVmax (red line) provides a superior net benefit compared to the model lacking SUVmax (blue line) across the most clinically relevant high-risk threshold probabilities.**


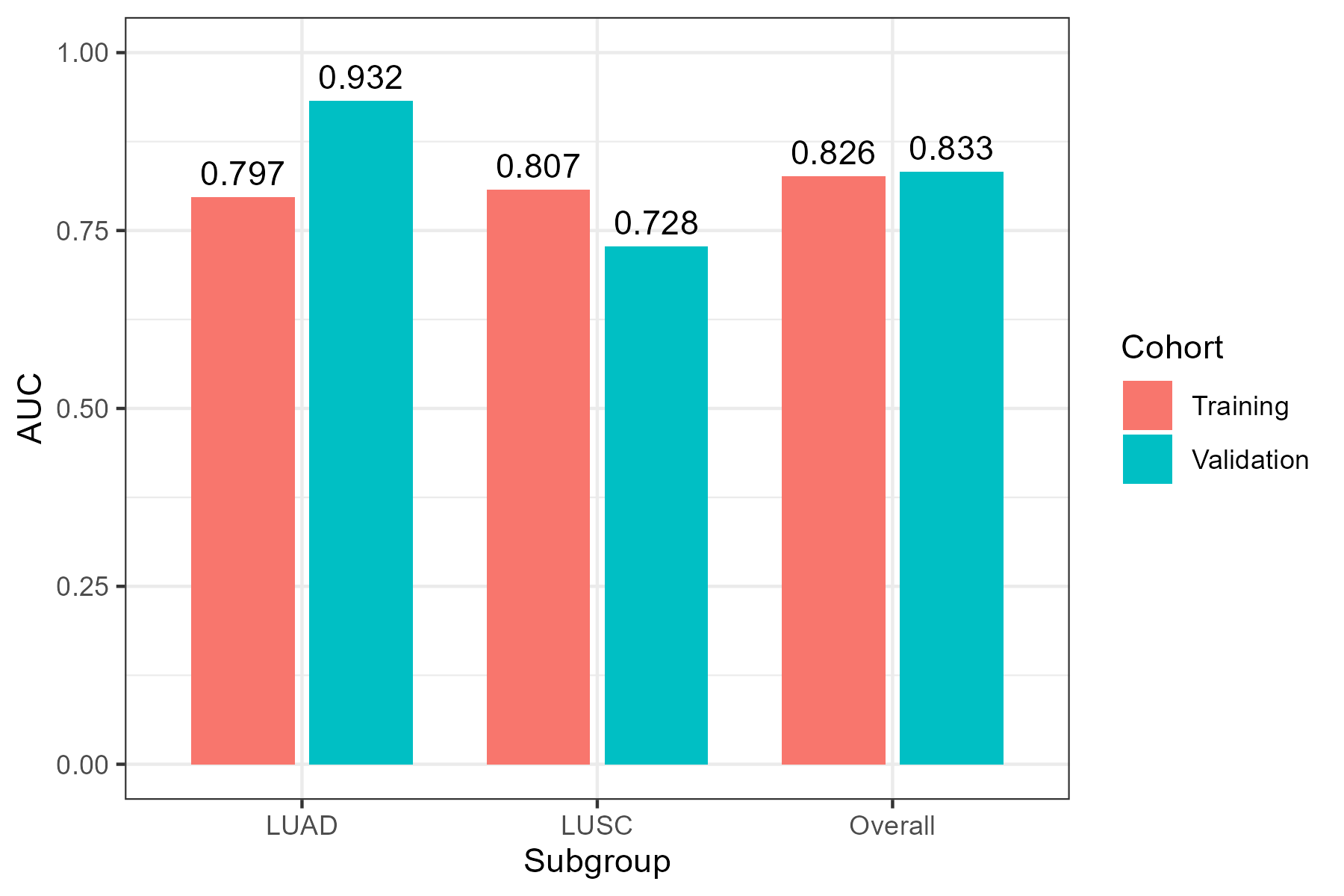


**Supplementary Figure S3. Subgroup analysis of the LR model across histological subtypes. AUC values for overall, LUAD, and LUSC subgroups in the training and external validation cohorts.**
